# Microenvironmental modulation of the developing tumour: an immune‐stromal dialogue

**DOI:** 10.1002/1878-0261.12773

**Published:** 2020-08-28

**Authors:** James O. Jones, William M. Moody, Jacqueline D. Shields

**Affiliations:** ^1^ MRC Cancer Unit Hutchison/MRC Research Centre University of Cambridge Cambridge UK; ^2^ Department of Oncology Cambridge University Hospitals NHS Foundation Trust Cambridge UK

**Keywords:** cancer‐associated fibroblast, extracellular matrix, immune, malignant transformation, stroma, tumour microenvironment

## Abstract

Successful establishment of a tumour relies on a cascade of interactions between cancer cells and stromal cells within an evolving microenvironment. Both immune and nonimmune cellular components are key factors in this process, and the individual players may change their role from tumour elimination to tumour promotion as the microenvironment develops. While the tumour–stroma crosstalk present in an established tumour is well‐studied, aspects in the early tumour or premalignant microenvironment have received less attention. This is in part due to the challenges in studying this process in the clinic or in mouse models. Here, we review the key anti‐ and pro‐tumour factors in the early microenvironment and discuss how understanding this process may be exploited in the clinic.

## Graphical abstract

Successful establishment of a tumour relies on a cascade of interactions between cancer cells and stromal cells within an evolving microenvironment. Both immune and nonimmune cellular components have key roles in this process. Here, we review the major anti‐ and pro‐tumour factors in the early tumour microenvironment and discuss how understanding this process may be exploited in the clinic.

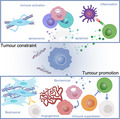

**Abbreviations**
Arg1: arginase 1
ATRA: all‐trans retinoic acid
CAF: cancer‐associated fibroblast
CC(L): C‐C motif chemokine (ligand)
CD: cluster of differentiation
Chi3L1: chitinase‐3‐like protein 1
CRC: colorectal cancer
CXCL: C‐X‐C motif chemokine
DAMPs: damage‐associated molecular patterns
DCs: dendritic cells
ECM: extracellular matrix
EGF: epidermal growth factor
FAP: fibroblast activation protein
FGF(R): fibroblast growth factor (receptor)
FSP1: fibroblast‐specific protein 1
Gas6: growth arrest‐specific 6
G‐CSF: granulocyte colony‐stimulating factor
GDF‐15: growth differentiation factor‐15
HGF: hepatocyte growth factor
HLA: human leucocyte antigen
HMGB1: high‐mobility group protein 1
HPV: human papillomavirus
ICAM‐1: intercellular adhesion molecule‐1
ICPI: immune checkpoint inhibitors
IDO: indoleamine dioxygenase
IFN: interferon
IGF: insulin‐like growth factor
IL: interleukin
ILCs: innate lymphoid cells
JAM2: junctional adhesion molecule 2
KPC: KrasLSL.G12D/+; p53R172H/+; PdxCretg/+
LAIR‐1: leucocyte‐associated immunoglobulin‐like receptor‐1
LIF: leukaemia inhibitory factor
LOX(L): lysyl oxidase (‐like proteins)
MAP(K): mitogen‐activated protein (kinase)
M‐CSF: macrophage colony‐stimulating factor
MDSCs: myeloid‐derived suppressor cells
MHC: major histocompatibility complex
MICA/B: MHC class I‐related proteins A/B
miR‐: microRNA‐
MMP: matrix metalloproteinase
MMTV‐PyM: mouse mammary tumour virus‐polyoma middle tumour antigen
MYL: myosin light chain
NET: neutrophil extracellular trap
NF‐kB: nuclear factor kappa‐light‐chain‐enhancer of activated B cells
NKG2D: natural killer group 2, member D
NKs: natural killer cells
NKTs: natural killer T cells
NOS: nitric oxide synthase
OAC: oesophageal adenocarcinoma
op/op: osteoporotic
OPN: osteopontin
P4HA1: prolyl 4‐hydroxylase a‐subunit isoform 1
PBMC: peripheral blood mononuclear cells
PD‐(L)1: programmed dead‐(ligand)1
PDAC: pancreatic ductal adenocarcinoma
PDGF(R): platelet‐derived growth factor (receptor)
PDX: patient‐derived xenograft
PGE2: prostaglandin E2
PLOD‐1, PLOD‐3: procollagen‐lysine 2‐oxoglutarate 5‐dioxygenase 1 and 3
PSC: pancreatic stellate cells
ROS: reactive oxygen species
SASP: senescence‐associated secretory phenotype
SHH: sonic hedgehog
SMA: smooth‐muscle actin
SPARC: secreted protein acidic and rich in cysteine
STAT: signal transducer and activator of transcription
TAM: tumour‐associated macrophage
TAN: tumour‐associated neutrophils
TAZ: transcriptional coactivator with PDZ‐binding motif
TCR: T‐cell receptor
TGF‐β: transforming growth factor beta
TH: T helper
TKI: tyrosine kinase inhibitor
TLR: toll‐like receptor
TNF: tumour necrosis factor
TRAIL: TNF‐regulated apoptosis‐inducing ligand
TRAMP: transgenic adenocarcinoma mouse prostate
T_reg_
: T regulatory cell
VEGF: vascular endothelial growth factor
XCL‐1: X‐C motif chemokine ligand 1
YAP: Yes‐associated protein

## 1 Introduction

The stepwise accumulation of genetic changes from healthy tissue through to malignancy, is accompanied by co‐option of neighbouring normal cells to support tumour development and progression. These ‘normal’ cells, which include immune and nonimmune populations, are collectively termed the stroma. Stromal cells and cancer cells collectively form the tumour microenvironment.

While much work has focused on the characterisation of oncogenic mutations within epithelial cells from the point of cancer initiation, the acquisition of mutations alone is not always sufficient to give rise to cancer, as the majority of mutagenic cells die or senesce [1, 2, 3]. Indeed, many premalignant lesions already bear a significant mutational burden; however, only a minority go on to develop cancer [4, 5, 6, 7] and considerable lag times from these initial mutations to tumour development have been reported [8]. This implies a role for the surrounding stromal environment from the point of cancer initiation, where non‐cell‐autonomous environmental cues provide a niche permissive for malignant transition.

With our increasing appreciation of the complexities of the tumour microenvironment, the concept of exploiting the stroma for therapeutic benefit is becoming more attractive. Thus, whether the characteristics of an evolving microenvironment can be exploited towards early diagnosis and patient stratification approaches in early stages of carcinogenesis remain‐ key questions to be addressed. In this review, we examine the state of our understanding of components within the microenvironment and their contribution to malignant transition. While immune components have been the subject of the most intense research, nonimmune constituents are now emerging as critical players, and here, we attempt to emphasise their role, highlighting emerging strategies to facilitate early detection.

## 2 Immune cell populations of the evolving tumour microenvironment: losing control

The recognition and elimination of genetically distinct transformed cells by the immune system (immunosurveillance) [9] represents a major barrier to the progression of early cancers. This is evident from numerous studies reporting a higher incidence of cancer in immunocompromised individuals [10, 11, 12, 13, 14] and in mice lacking key cytotoxic components of the immune systems such as IFN‐γ, perforin and natural killer cells (NK cells) [15, 16]. The potential of the immune system to eliminate established tumours in humans has been well‐demonstrated by the effectiveness of immune checkpoint inhibitors (ICPIs). Therefore, the immune system's role in interacting with virtually all nonimmune components of the TME as well as mediating signalling beyond the physical confines of the TME to the tumour macroenvironment has led to great interest in the role of the immune system in early tumorigenesis [17].

The presence of immune cells in tumours capable of recognising mutated cancer cells raises several important questions, which until now remain a significant challenge in oncology: How do cancer cells avoid destruction by the immune system? How is immune control lost? And when during tumorigenesis is this control lost?

### 2.1 Adaptive immunity in malignant transformation

T lymphocytes are key determinants in tumour fate. Elevated CD8 infiltration and high CD8:T_reg_ ratios in established tumours are associated with favourable long‐term prognosis [18, 19, 20, 21]. In early lesions experiencing active immune surveillance, tumour neoantigens generated as a result of genetic instability are presented on MHC, alerting T cells to the presence of cancer cells leading to rapid elimination [22, 23, 24, 25, 26]. Studies using carcinogen‐driven murine models have shown that the premalignant microenvironment is significantly more immune stimulatory than established tumour microenvironments, supporting potent T‐cell activation [27, 28]. In genetically engineered mouse models, DuPage *et al*. [22] elegantly demonstrated antigen‐dependent accumulation of T cells was sufficient to delay malignant lung tumour progression. Premalignant lesions have increased numbers of T cells compared with fully developed invasive lesions, and those with lower infiltration exhibit higher incidence of progression to cancer, indicating a key relationship between T cells and the epithelium in this stepwise process [28, 29, 30, 31, 32, 33].

However, this is in stark contrast to established tumours which frequently present with a paucity of tumour‐infiltrating lymphocytes exhibiting an impaired cytotoxic capacity. Established tumours escape immune attack through four major mechanisms. First, mutation of constituents of the antigen presentation pathway reduces neoantigen presentation rendering cancer cells ‘invisible’ to T cells. Indeed, 40% of human non‐small‐cell lung cancers exhibit defects in human leucocyte antigens (HLAs), reducing antigen presentation and promoting immune escape [34]. Second, functional T cells eliminate immunogenic clones promoting the development of less heterogeneous, less immunogenic tumours in the process, termed immunoediting. In murine sarcoma, antigen‐specific T cells present in early lesions were critical for immunoediting to permit outgrowth of less immunogenic clones [35]. Third, tumours induce a state of dysfunction in infiltrating T cells often termed ‘immune exhaustion’. DuPage *et al*. [22] showed that despite an initially effective T‐cell response to delay lung tumour progression, T cells did not persist and exhibited a decreased cytotoxicity, despite continued antigen exposure. Indeed, it has been shown that persistent antigen exposure is a critical trigger for initiation of the T‐cell exhaustion signature [36, 37], which is increasingly appreciated as a graded spectrum of inactivity with its origins in the early TME. In other models examining loss of T‐cell function in premalignant lesions, increases in PD‐1/PD‐L1 expression, T_reg_ diversity and numbers of circulating T_regs_ were indicative of T‐cell dysfunction [31, 38, 39]. Fourth, established tumours develop an immune‐suppressive microenvironment which protects them from T‐cell‐mediated immunity. This has been historically demonstrated by the phenomenon of ‘concomitant immunity’. Tumour formation is induced in a mouse. Following excision of the tumour, the same mouse demonstrates immune‐mediated resistance to rechallenge with cells from its own tumour. This is an elegant demonstration that the immune system may eliminate isolated tumour cells, but once the microenvironment is formed the tumour is protected from circulating immunity [40]. Within the evolving TME, transforming growth factor beta (TGF‐β), epithelial or immune derived, can further amplify loss of T‐cell‐mediated immune surveillance [41, 42]. T_regs_ suppress T‐cell effector functions [43, 44], downregulating genes involved in the cytotoxic function of CD8 T cells including granzyme B and IFN‐γ, ultimately tipping the immune balance towards tolerance and facilitating immune evasion. TGF‐β is key to the initiation of regulatory programmes and differentiation of CD4 T cells into T_regs_ [41, 45, 46, 47, 48]. Interestingly, during malignant transition TGF‐β operates in a context‐specific manner, playing opposing roles, initially functioning as a tumour suppressor capable of inducing apoptosis in premalignant cells before behaving as a potent immune suppressor, reviewed extensively elsewhere [49, 50, 51]. The wider immune functions of other microenvironment components are discussed in more detail later in this review. Thus, the perturbation of T‐cell functionality in premalignant lesions does not occur in isolation, but via crosstalk with other constituents of the immune milieu.

Collectively, these reports add to a growing body of evidence suggesting that while T cells are essential for immune control of premalignant lesions, loss of T‐cell‐mediated immune surveillance may occur prior to malignant transition and that immune involvement actively promotes progression to cancer [31, 38, 52].

### 2.2 Innate modulation of the transforming tumour microenvironment

Although critical fate determinants, T cells do not operate in isolation, engaging in extensive crosstalk with other immune cells within the developing microenvironment [32]. The innate immune system, comprising neutrophils, NK cells, innate lymphoid cells (ILCs), dendritic cells (DCs) and macrophages, functions primarily as a rapid response to pathogens. In the tumour context, an initially protective response switches to promote carcinogenesis. While directly contributing to tumorigenesis by selecting immunogenic clones, the innate immune compartment ultimately shapes the formation of a tumour microenvironment providing inflammatory cues capable of controlling T‐cell fate, modulating mutated cancer cells themselves and modifying underlying tissues.

#### 2.2.1 Macrophages

Of all immune cells, macrophages are the most abundant and display arguably the highest plasticity, responding rapidly to a diverse array of environmental stimuli. Activated pro‐inflammatory (M1) and anti‐inflammatory (wound healing, M2) populations have been described extensively [53, 54]. However, in the context of a tumour, M1 and M2 nomenclatures represent a grossly oversimplified classification, with macrophages existing along a spectrum of phenotypes from pro‐ to anti‐inflammatory depending on the localised cues they receive [55, 56, 57, 58, 59]. Indeed, reflecting the breadth of diversity of tumour‐derived cues, studies have described macrophages expressing both markers typical of M1 and M2 phenotypes, and traits overlapping with myeloid‐derived suppressor cells (MDSCs). Heterogeneity within the microenvironment of a developing tumour further amplifies intratumoral macrophage diversity and diversity across cancer types.

In very early stages of tumour development, enhanced numbers of macrophages have been observed [32, 60], and those recruited exhibit a pro‐inflammatory phenotype, activated by host factors such as DAMPs and tumour cell DNA [24, 61, 62]. Such infiltrates contribute to immune surveillance and are capable of directly eliminating immunogenic tumour cell clones via release of toxic mediators such as TNF, interleukin‐2 (IL‐2), reactivate nitrogen and oxygen species [63, 64, 65, 66, 67]. This behaviour is reminiscent of their role in infection, phagocytosing and presenting tumour‐derived antigen to incoming CD8 T cells [68, 69, 70, 71, 72]. Moreover, augmenting these effects, macrophage‐derived IL‐1 activates surveilling innate and adaptive immune cells to inhibit malignant progression. As occurs for T‐cell surveillance, tumours evolve strategies to evade immune control. Recent work has identified a mechanism used by neoplastic cells to avoid macrophage‐mediated immune surveillance during the early stages of tumorigenesis. Epithelial‐derived GDF‐15 suppressed macrophage cytotoxic activity by inhibiting TNF and NO production in an NF‐κB‐dependent manner [73]. Therefore, in response to a rapidly adapting cytokine milieu within developing lesions including TGF, IL‐10, IL‐4 and M‐CSF, macrophages acquire a tumour‐promoting, immune‐suppressive phenotype, remodelling the microenvironment in the process [74, 75, 76, 77].

However, these antitumour effects of very early lesions are replaced by a tumour‐promoting phenotype at an early stage of carcinogenesis. This requirement for macrophages in malignant progression was elegantly demonstrated by Lin *et al*. who crossed MMTV‐PyMT mice, which develop breast tumours spontaneously, with osteoporotic (op/op) mice lacking CSF‐1, a cytokine critical for macrophage function. While they observed little impact on the development of premalignant lesions, progression to invasive carcinoma was significantly impaired [76, 78].

A key feature of once antitumour, inflammatory macrophages is the acquisition of immune‐suppressive traits to facilitate malignant progression [60, 76, 79, 80]. To support immune suppression, and thus evasion of early immune surveillance, macrophages use both direct and indirect mechanisms. Expression of immune checkpoints such as PD‐L1 inhibits T‐cell function, and release of suppressive cytokines, metabolites and proteases has far‐reaching effects on the immune compartment [81, 82]. Pro‐inflammatory cytokines such as IL‐12 are replaced with the secretion of IL‐10 [82, 83], Indoleamine dioxygenase (IDO) [20, 84] and TGF‐β [42, 49, 82, 85, 86], which together impair T‐cell effector activity [82, 87, 88] and promote induction of T_reg_ phenotypes [47, 89]. CCL22 recruits T_regs_ and along with CCL5 and CCL17 can inhibit T‐cell proliferation [90, 91]. Similarly, IL‐10 and TGF‐β inhibit DC maturation, reducing antigen‐presenting capacity and downstream T‐cell responses.

Macrophages within the premalignant microenvironment may also elicit tumour‐promoting activities beyond influencing immune cells, either via direct effects on epithelial cells or by indirect effects on other nonimmune stromal populations. There are several direct epithelial mechanisms. First, factors such as ROS and NOS also induce DNA damage contributing to genomic instability and mutational burden [74, 75, 92, 93, 94]. Second, the release of cytokines and growth factors such as EGF stimulates epithelial proliferation [95, 96, 97, 98]. In both chronic inflammation and Kras‐driven models of pancreatic cancer, macrophages were shown to drive metaplasia of acinar to ductal cells. Pancreatic acinar cells with oncogenic *KRASG12D* upregulated the expression of ICAM‐1 to recruit macrophages, which through matrix metalloproteinase‐9 (MMP9) and TNF‐α production induced metaplasia, the earliest abnormal pancreatic lesions [60]. Also in pancreatic cancer, reshaping of the premalignant microenvironment to a PDAC phenotype has been reported to require early recruitment of macrophages through CCL9 and CCL2 [99]. Macrophage‐derived Gas6 has also recently been described as a critical regulator for the transition between premalignant breast cancer and invasive breast cancer [100]. Signalling via Axl on premalignant epithelial cells, having engaged with macrophage‐derived Gas6, induced downstream survival signals and concurrent loss of E‐cadherin.

Beyond their direct influence on epithelial cells, as for any organ, an oxygen supply needs to be established to support an expanding epithelial compartment in hyperplastic lesions [101, 102, 103]. Here, macrophages, including a subset expressing Tie2, contribute to the angiogenic switch and function as potent inducers of angiogenesis via production of diverse pro‐angiogenic factors VEGF, TNF‐α, IL‐8, MMP9 and FGF‐2 [104, 105, 106, 107, 108, 109]. These features have been demonstrated in transgenic mouse models which recapitulate the very early neoplastic lesions. In an inducible mouse model of preneoplastic changes in the mammary epithelium, macrophage recruitment was necessary for epithelial proliferation and induction of angiogenesis [110]. Similarly, in mice null for CSF1, delayed progression from premalignant to invasive lesions was due a failure to induce angiogenesis by macrophages [78, 111], a process dependent on MMP9 [112]. This was true also for intestinal tumorigenesis [113]. Moreover, macrophage‐derived MMP9 plays a pivotal role in tumour angiogenesis, regulating the bioavailability of VEGF [114, 115].

Macrophages are also involved in remodelling of the underlying tissues. The extracellular matrix (ECM), a 3D network composed of polysaccharides, proteins including collagens and glycoproteins, provides biochemical and biomechanical cues critical for tumour progression [116, 117]. Macrophages are frequently observed within collagen‐rich areas of developing tumours [118] where they are implicated in the deposition of matrix [86, 119, 120, 121, 122, 123, 124], fibril organisation [119, 125, 126, 127], cross‐linking (stiffening the ECM) and degradation to release growth factors and support cell motility [120, 128]. Here, they have been shown to upregulate expression of the matricellular glycoproteins, osteopontin, osteoactivin, fibronectin, collagen types I VI and XIV and secreted protein acidic and rich in cysteine (SPARC) needed for the assembly and organisation of collagenous matrix [119, 129, 130, 131]. Macrophages produce enzymes to modify collagen assembly such as prolyl 4‐hydroxylase a‐subunit isoform 1 (P4HA1) and procollagen‐lysine 2‐oxoglutarate 5‐dioxygenase 1 and 3 (PLOD‐1, PLOD‐3) [119], and modify secreted structure via expression of lysyl oxidase (LOX) and lysyl oxidase‐like (LOXL) proteins [126], supporting matrix stiffening needed for changes in epithelial morphology (discussed in detail later).

#### 2.2.2 ILCs and NK cells

Within the early cancer setting, ILCs are extremely efficient at eliminating malignant cells through the expression of perforin and granzyme, as well as death ligands, such as TNF‐regulated apoptosis‐inducing ligand (TRAIL) and Fas ligand [132, 133, 134] ILCs comprise the cytotoxic NK cells, and the more recently discovered ‘helper’ ILC subsets. NK cells are key participants in immune surveillance of early lesions, ‘recognising’ malignant cells by the absence of MHC I on cancer cells evading cytotoxic T cells [135], and hence, their presence is associated with good prognosis. Evidence supporting this antitumour role came from studies showing increased cancer incidence in mice and humans lacking NK cells [136, 137], and increased cancer risk in patients with low NK activity [138].

A fluctuating balance of activating and inhibitory receptor–ligand interactions on NK cells controls their activity. The most interesting of these, from a tumour immunology perspective, has been the natural killer group 2, member D (NKG2D) receptor, which can recognise MHC class I‐related proteins A and B (MICA and MICB), ectopically expressed antigens such as Rae1 or H60, as well as DNA damage‐induced cell surface ligands [139, 140], facilitating tumour cell elimination [140, 141, 142]. Cytokines including IFN‐γ, IL‐12, IL‐18 and IL‐15, and the cell surface adhesion molecule LFA‐1 within the TME also potently activate NK cells [143, 144]. Activated NK cells produce TNF, IFN‐γ or CSF‐2, modulating leucocyte function to amplify antitumour activity, and also express chemokines, such as CCL5 and XCL1 to recruit DCs [145, 146]. As the TME establishes, however, NK cells become much less effective at killing their targets, exhibiting decreased cytotoxicity and inflammatory cytokine production. To evade destruction by NK cells, tumours employ both direct and indirect mechanisms, including coating themselves in collagen and platelets to shield themselves from NK detection [147, 148]. Cytokines also add to their newly acquired suppressive phenotype, weakening cytotoxic capacity and arresting T‐cell proliferation [145, 149, 150, 151, 152].

The role of the other ILC populations is poorly understood and varies depending on the local microenvironment, with both immune‐protective and tumour‐promoting effects demonstrated in lung, skin and colon cancer [153, 154, 155]. ILCs are largely tissue‐resident and divided into ILC1, ILC2 and ILC3 subsets functionally corresponding to the TH1, TH2 and TH17 subsets of CD4^+^ T cells – however this is a considerable simplification, and the full nature of these subsets' functions is still under investigation. Analogous to NK cells, ILC1s and ILC3s are also activated by IL‐15, and contribute to immune surveillance through the release of TNF, IL‐8 and IL‐2, promoting leucocyte recruitment and proliferation [156, 157]. During malignant progression, however, within an increasingly TGF‐β‐rich environment, NKs have been reported to convert into ILC1‐like cells with a reduced ability to control tumour growth [145, 149]. ILC3s have also been shown to drive epithelial proliferation in a IL‐22‐dependent manner [158].

Another T‐cell population with innate‐like properties are the natural killer T cells (NKTs). These are a unique population of T cells that recognise lipid antigens presented on the class I‐like molecule C1d. ‘Type I’ NKTs use a semi‐invariant TCR made of a specific alpha chain with a small number of beta chains and respond *in vitro* to the lipid alpha Gal‐Cer. Mice lacking type I NKTs have generally been found to have defects in tumour immune surveillance and there is interest in using alpha Gal‐Cer to stimulate antitumour immunity, though results in patients have been limited. However, in a transgenic model of intestinal polyps (‘APC min’ mouse), type I NKTs were found to promote polyp formation by recruitment of T_regs_. ‘Type II’ NKTs (or ‘nonclassical’ NKTs) use diverse alpha and beta TCR chains; in contrast to type I, mouse experiments suggest they have an immune‐regulatory, tumour‐promoting function. Like the ILCs, the importance of NKTs in early tumorigenesis is not well‐understood.

#### 2.2.3 Neutrophils

Neutrophils operate as vital first responders during infection. Given this critical early role in infection, it follows that neutrophils also contribute extensively to early tumorigenesis, with anti‐ and pro‐tumour activities reported. As for macrophages, this may in part be a consequence of their functional plasticity. Indeed, the phenotype of neutrophils in the TME depends on tumour type and stage. In early tumorigenesis, neutrophils are recruited by epithelial‐ and stroma‐derived chemokines such as G‐CSF, CXCL8, CXCL1, CXCL2, CXCL3 and CXCL5 [159, 160, 161]. Here, they elicit antitumour activity through the release of cytotoxic granules, production of TNF‐α, NO, H_2_O_2_, TRAIL and IFN‐γ and expression of costimulatory molecules [162, 163, 164]. At this stage, skewed by IFN‐β, tumour‐associated neutrophils (TANs) attract and promote activation of surveilling T cells [164, 165, 166].

However, directly contradicting this antitumour activity, TANs have also been reported to contribute to carcinogenesis from the point of tumour initiation. Local TGF‐β pushes neutrophils towards a tumour‐promoting phenotype [166, 167], inducing genetic instability [168] and MAPK‐dependent proliferation of preneoplastic cells [169, 170] as well as releasing pro‐inflammatory cytokines [97]. In particular, neutrophil‐derived IL‐6 and IL‐11 have been shown to promote cancer cell proliferation and inhibit apoptosis via STAT3 [171]. Now, TANs preferentially recruit T_regs_, directly suppressing CD8 T cells via PD‐L1 and indirectly by IL‐8‐driven arginase production [172, 173, 174, 175]. Once established, TANs contribute to shaping of the microenvironment inducing angiogenesis, remodelling of ECM through deposition of neutrophil elastase and MMP release [176, 177, 178, 179]. A further feature of neutrophils is the production of neutrophil extracellular traps (NETs). These extracellular DNA structures are decorated in cytotoxic granules, MMPs and neutrophil elastase, and although a growing body of evidence describing roles in tumour circulation [180], metastatic colonisation [181] and tumour dormancy [182] exists, their role in early stages of primary tumour development remains sparse [183].

#### 2.2.4 Dendritic cells

Dendritic cells are professional antigen‐presenting cells that bridge the innate and adaptive immune compartments, stimulating T cells in an antigen‐specific fashion. In the still inflammatory early TME, proficient DCs effectively present tumour antigen to T cells alongside costimulatory molecules CD40, CD80 and CD86, thereby playing a central role in immune surveillance [67, 184, 185, 186]. Here, their presence is associated with good prognosis [146, 184]. However, exposure to suppressive modulators such as VEGF, IL‐10, TGF‐β and prostaglandin E2 (PGE2) and microenvironmental cues such as accumulation of lipids and decreased pH inhibits DC maturation, decreasing expression of costimulatory modules and hence reducing antigen presentation capacity [82, 187, 188, 189, 190]. This renders DCs less capable of priming T cells, while more prone to promote TH2 and T_reg_ responses [191, 192, 193]. PD‐L1 expression by DC directly suppresses infiltrating CD8 T cells [186, 194]. Cytokine‐induced changes in transcriptional and metabolic pathways further promote a tolerogenic phenotype, stimulating expression of factors such as IDO, Arg1, iNOS and STAT3 [195, 196, 197, 198, 199]. To further complicate matters, with reports of anti‐ and pro‐tumour activities depending on the site examined, our understanding of DCs may be confounded by tissue specificities in addition to stage of development [191, 200].

**Fig. 1:**
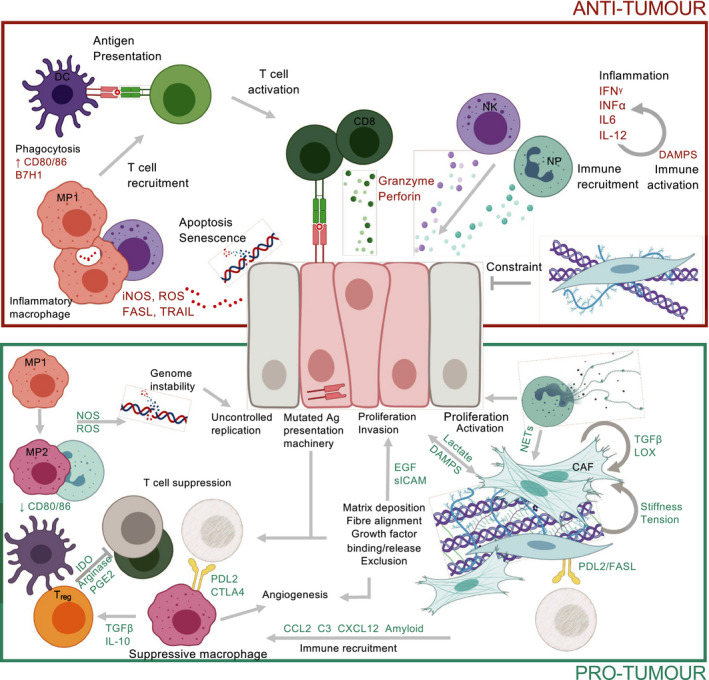
Functions of the early tumour microenvironment. Recruitment and activation of cytotoxic CD8 T cells, NK cells, neutrophils and macrophages result in the elimination of mutated epithelial cells. However, these initial antitumour events driven by immune surveillance give way to a suppressive environment with tumour‐promoting functions. Extensive crosstalk between stromal populations and cancer cells elicit and amplify pro‐tumour functions. These include fibroblast activation and ECM remodelling; induction of the angiogenic switch; release of growth factors and intermediates to support genomic instability and proliferation; immune exclusion; collaboration between immune cells to prevent antitumour responses including impaired antigen presentation, reducing cytotoxic capacity; release of suppressive cytokines; and T‐cell deletion/induction of anergy. CD8, cytotoxic T cell; T_reg_, regulatory T cell; MP1, inflammatory macrophage; MP2, suppressive macrophage; DC, dendritic cell; NP, neutrophil; NK, natural killer cell.

Thus, although our knowledge is increasing, the role of the immune infiltrate in premalignant tissues and the balance of protection vs. progression remains unclear (Fig. 1). With a large body of correlative data, detailed phenotypic and functional analyses are required. Furthermore, as discussed later in this review, the choice of model to study the microenvironment in carcinogenesis is critical. Of note, correlations between inflammation and progression of premalignant states to cancer are stronger along the gastrointestinal tract than other sites. These include Barrett's oesophagus, Crohn's disease and ulcerative colitis. This raises the questions of whether different anatomical sites exhibit different immune infiltration, whether tissue‐specific responses to inflammation exist, and how these may contribute to progression of precancerous lesions at different sites. The pro‐tumour functions of the innate immune system in established tumours have been particularly well‐studied, but this in part reflects the technical challenges of mechanistic studies of very early lesions.

The mechanisms underlying the transition from early immune surveillance by innate immune cells to tumour‐promoting phenotypes of macrophages, NK cells, neutrophils and DCs, later during tumorigenesis, are poorly understood. This is in part due to the technical challenges of studying very early lesions in a ‘true’ epithelial setting, which requires transgenic mouse models. Much of the evidence for early innate immune surveillance comes from macroscopic observations of increased cancer rates in immune‐suppressed states, rather than direct observation of the premalignant epithelium. One could hypothesise two key reasons for this shift. According to a cancer cell centric view, premalignant lesions that acquire the ability to co‐opt pro‐tumorigenic innate immunity can grow into tumours [201, 202]. Notably, cancer presents a paradox to the standard alarm signal sensors of the immune system: although tumours are genetically different and, thus, capable of promoting antigenic stimulation of T cells, they also release endogenous danger signals known as damage‐associated molecular patterns (DAMPs) to alert the innate immune system. DAMPs tend to generate an innate response compatible with immune regulation, T‐cell suppression and healing. The latter involves the secretion of epithelial growth factors and angiogenic signals which both support tumour growth. Indeed, tumours have been famously likened to ‘wounds that do not heal’ [203]. From a wider point of view, this makes sense, as the innate system has evolved to protect damaged epithelial cells under normal circumstances. Importantly, the inflammatory insult often precedes the premalignant transformation. It is well known that chronically inflamed nonmalignant pathologies such as ulcerative colitis are at high risk of generating malignant lesions, because the inflammatory microenvironment provides an ideal context for premalignant lesions to ‘flourish’ into an invasive cancer [204].

## 3 Stroma in malignant transformation: landscaping the tumour microenvironment

**Fig. 2:**
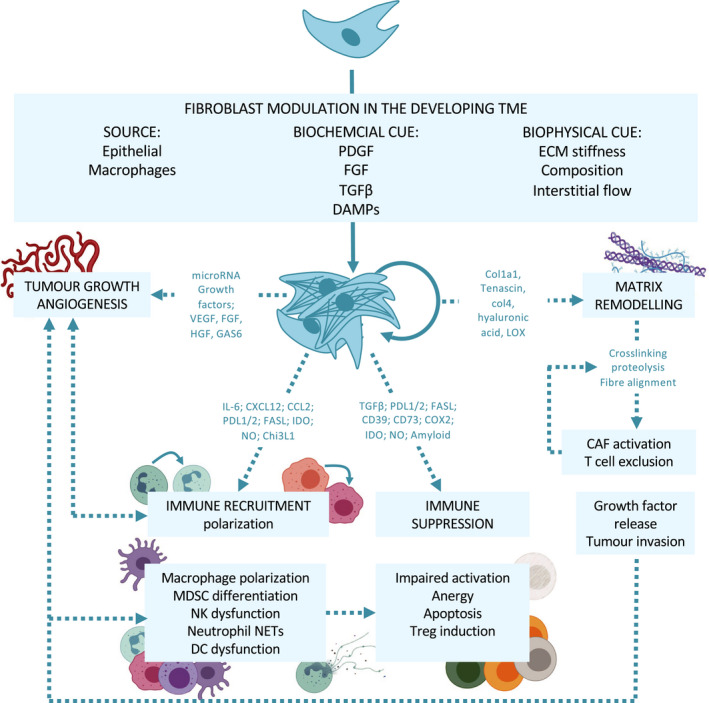
Fibroblast adaptation in the early tumour microenvironment. Within a rapidly changing environment, local cues either biochemical or biophysical support accumulation and further alterations to an already heterogeneous compartment. With few reports of antitumour activity, most CAF traits reported across known subsets are pro‐tumour. These include release of factors to support cancer cell proliferation and the angiogenic switch, cytokines to modulate immune recruitment, polarisation, suppression and ECM remodelling. CAF‐induced matrix remodelling operates on multiple levels, binding and releasing cytokine sinks, excluding immune cells and altering functionality, supporting cell migration and further activating CAFs in a feedforward loop.

While the interactions between malignant cells and the immune system play a key role in establishing the tumour microenvironment during malignant transition, other components of the host tissue play a critical and historically overlooked part. Indeed, the accumulation of stromal components including fibroblasts and vessels is also reported in premalignant lesions including oesophageal, pancreatic and skin cancers [205, 206, 207, 208, 209, 210]. Here, we will briefly discuss emerging roles and our current understanding of these populations to carcinogenesis (Fig. 2). Cancer‐associated fibroblasts (CAFs) are one of the most abundant stromal components in the developing TME and are recognised by numerous markers such as fibroblast‐specific protein 1 (FSP1/S100A4), vimentin, α‐smooth‐muscle actin (αSMA), fibroblast activation protein (FAP), PDGF receptor‐α (PDGFR‐α) and podoplanin. CAFs largely originate as a result of the chronic wound‐healing response present in the early TME and display a genomic, epigenomic and secretomic profile distinct from untransformed fibroblasts [211, 212, 213].

Cancer‐associated fibroblasts exhibit diverse pro‐tumour functions including growth factor secretion, angiogenesis, immunoregulation [214, 215, 216, 217], ECM remodelling [218, 219] and promoting cancer stemness [220], invasion [221, 222, 223] and chemoresistance [216, 224, 225, 226, 227]. To enable such diversity, the CAF compartment is highly heterogeneous, composed of multiple subpopulations displaying functional specialisation from potentially different origins [228, 229, 230, 231]. However, observations of fibroblast accumulation and expansion around premalignant lesions prior to malignant transformation indicate that, at initial stages, fibroblasts are predominantly tissue‐resident [207, 229]. Although the majority of studies have cited pro‐tumour effects of CAFs, pancreatic cancer studies using the KPC model have yielded more contradictory results, highlighting specific roles of CAF subsets. Here, Feig *et al*. [216] depleted CAFs based on expression of FAP, reducing tumour burden. In contrast, studies ablating αSMA CAFs resulted in poorly differentiated tumours and shortened survival implying that αSMA‐expressing CAFs, at least in early pancreatic lesions, exhibit tumour‐suppressive activity mediated via immune activation and physical constraint [232, 233]. Together, with observations of CAF accumulation in early lesions, these data indicate that the initial fibroblast response to mutated cells can be tumour‐suppressive, before conversion to a tumour‐promoting phenotype [234, 235]. That said, fibroblasts developing with premalignant lesions can significantly influence adjacent epithelial cells via paracrine signalling [210, 220, 236, 237, 238, 239]. Fibroblast‐derived factors including HGF, IGF, EGF, TGF‐β, IL‐6, LIF, oncostatin M, FGF and metabolites elicit both transformative and proliferative effects on their targets [236, 240, 241, 242]. Early *in vitro* and xenograft studies in prostate cancer showed that tumour fibroblasts but not normal counterparts could stimulate proliferation and transformation of epithelial cells from benign prostate hyperplasia. Interestingly, CAFs alone were unable to induce growth of normal epithelium, providing early indications of a requirement for reciprocal communication between the two compartments for malignant transformation to occur. Indeed, epithelial cells in precancerous states have been shown to use TGF‐β, PDGF‐D and FGF‐2 to recruit, activate and transform tissue‐resident fibroblasts towards a CAF phenotype [234, 235]. And, while genetically stable, epigenetic alterations and changes in expression of noncoding RNA underpin many gene expression changes following the development of CAF traits [243, 244, 245, 246]. For example, changes in miR‐31, miR‐214 and miR‐155 facilitate conversion of normal fibroblasts into CAFs, whereas the upregulation of miR‐21 impacts TGF‐β signalling to support acquisition of a CAF phenotype.

Increasing genomic stress can promote the production of activin and TGF‐β, supporting CAF activation [247]. Similarly, the release of ‘danger’ signals such as IL‐1α or DAMPs by epithelial cells is sufficient to promote CAF activation and upregulation of pro‐inflammatory genes, with IL‐1β for example, released to promote epithelial proliferation [248, 249, 250]. Physical stresses created in a rapidly changing environment also support formation and expansion CAFs [218]. For example, responses to environmental stiffness via YAP‐TAZ signalling and heat shock factor 1 have been reported to reprogramme fibroblasts towards a more tumour‐promoting phenotype [251, 252]. In response to epithelial‐derived factors, CAFs may proliferate, become ‘activated’ or become senescent [253], a process which itself can exert significant effects to adjacent cells via the senescence‐associated secretory phenotype (SASP; reviewed extensively in Ref. [238, 254]).

Cancer‐associated fibroblasts exhibit a capacity to modulate lymphatic vessels, their presence correlating with lymphatic vessel density and metastasis [255]. Here, CAF‐secreted factors such as hyaluronan [256], FGF, HGF and VEGF‐C directly induce lymphangiogenesis [257, 258, 259], while tumour‐derived lysyl oxidase‐like protein 2 (LOXL2) and sonic hedgehog have been reported to stimulate CAFs to upregulate VEGF‐C and SDF‐1α to support lymphatic expansion [260, 261]. CAF‐derived ECM components such as hyaluronan, fibronectin, collagen, laminin and osteopontin have further been identified as lymphangiogenic drivers [257, 262], as have CAF‐derived microvesicles which are reported to exert pro‐lymphangiogenic effects via angiopoietin and Tie2 driving VEGFR3 expression on lymphatics [263]. Whether similar communications are established in the early TME to support malignant transition remains to be determined.

### 3.1 Crosstalk of CAFs with immune cells

Cancer‐associated fibroblasts are emerging as key immune modulators in the tumour context, with increasing evidence to suggest that crosstalk between the nonimmune stroma and leukocytes is essential for escape from early immune control and tumour progression [205, 214, 215, 264, 265, 266]. CAFs contribute to initial tumour growth via promotion of an initial local inflammatory microenvironment through upregulation of NF‐κB‐controlled pathways in response to IL‐1β produced by tissue‐resident immune cells in the skin [205]. This gene signature was observed in the preneoplastic ‘hyperplastic’ stage, suggesting that early CAF transformation is critical for both the induction and maintenance of tumour‐promoting inflammation in the skin. Intriguingly when CAFs from a similar time point in cervical cancer were examined, no such gene signature was found, hinting at the importance of the tissue specificity of both the fibroblastic origin and the resident immune microenvironment when driving early‐stage tumour progression.

Immune‐modulatory fibroblasts within the developing tumour microenvironment can impact recruitment, retention and polarisation of the innate immune compartment [183, 205, 249, 266, 267, 268, 269]. We have shown that in early stages of cancer, immune‐regulatory CAFs recruit macrophages through a variety of signals including complement C3, CSF and CXCL12 [269] and others have shown similar to CXCL12 and CXCL16, and via the CCL2–CCR2 axis [270, 271, 272, 273]. Functioning as potent modulators of macrophage behaviour [205, 265, 267, 272], fibroblast‐derived chitinase‐3‐like protein 1 (Chi3L1) also drives macrophages towards a suppressive phenotype in mammary lesions [266] as does CXCL12 in prostate cancer [270]. Similarly, pancreatic stellate cells (PSCs) isolated from human pancreatic tumours, but not healthy tissue, were able to induce an MDSC phenotype in peripheral blood mononuclear cells (PBMCs) in an IL‐6‐dependent manner [274]. CAFs also upregulate TLRs, enabling them to respond to environmental triggers such as DAMPs and release‐soluble mediators that support both tumour proliferation and immune suppression during tumorigenesis [250, 275, 276]. This impact, however, appears to be tissue‐ and TLR‐specific, with TLR7 and TLR9 instead promoting effective immune responses required for immune surveillance [277, 278, 279].

Notably, mounting evidence supports a role for CAFs in modulation of adaptive immune components, shaping T‐cell dysfunction associated with loss of immune surveillance. The production of soluble factors such as IL‐10 and TGF‐β, and metabolic mediators prostaglandin E2 (PGE2), indoleamine 2,3‐dioxygenase (IDO) and arginase can impair activation and cytotoxicity or push responses towards tumour‐promoting TH2 and TH17 responses [280, 281, 282, 283]. FAP‐expressing CAFs can modulate T cells in an NO‐dependent manner, their depletion resulting in increased CD8 T‐cell tumour infiltration [215], and podoplanin expressing CAFs cross‐presented tumour antigen in MHC I to directly induce antigen‐specific antigen‐dependent T‐cell deletion and anergy via PD‐L2 and Fas interactions [214]. Recently, a population expressing MHC II and CD74 has also been identified implying a similar capacity to modulate CD4 T cells [217]. However, indicative that these suppressive behaviours develop as the microenvironment becomes more established, and CAFs may initially elicit T‐cell stimulatory effects, reports have also suggested a role for CAF‐derived IL‐6‐activating T‐cell production of IFN‐γ and IL‐17A [281]. Moreover, CAF‐driven recruitment and differentiation of T_regs_ contribute to the onset of an immune‐suppressive environment [46]. In human breast and ovarian cancers, subpopulations of CAF were able to recruit T_regs_ via CXCL12, mediate retention by OX40L, PD‐L2 and JAM2 interactions and support survival [284, 285]. CAFs also exhibit the capacity to disrupt NK function either by abolishing production of cytotoxic granules, granzyme B and perforin by PGE2 and IDO, or by secretion of MMPs to cleave NKG2D ligands on target tumour cells [286, 287, 288]. While these studies were performed in metastatic lesions, such behaviours may also apply to the loss of antitumour activity contributing to immune evasion during malignant transition.

As alluded to previously, a complex interplay exists between CAFs and immune cells, the generation of the tumour microenvironment relies on reciprocal signals. For example, while CAFs impact macrophages and neutrophils, neutrophils also release ROS and NETs to deliver pro‐fibrotic signals [183, 289]. Likewise, CAFs drive macrophage recruitment and polarisation, yet IL‐4‐polarised suppressive macrophages induce expression of the collagen cross‐linker enzyme lysyl hydroxylase 2 in adjacent fibroblasts to promote stiffening of the ECM [281, 290].

### 3.2 CAFs and the extracellular matrix

Following tissue damage, fibroblasts of healthy tissues rapidly synthesise and deposit ECM as part of the wound‐healing response. In cancer, however, these mechanisms are perturbed to generate a fibrotic, permissive niche [117, 123, 291]. Here, fibroblasts serve as the dominant source of ECM and remodelling enzymes, producing amongst others, collagen family members, laminins, tenascin C, fibronectin, versican, hyaluronic acid, lysyl oxidase (LOX) family members and MMPs [292, 293, 294, 295, 296]. The deposition of ECM, even at very early stages of tumorigenesis, changes the mechanics of a tissue, contributing to transition from premalignant disease to cancer [206, 297, 298]. For example, in breast cancer, a collagen‐dense microenvironment frequently presenting as high mammographic density represents a significant risk factor. This was confirmed when fibroblasts derived from healthy women with high mammographic density (but no breast cancer) exhibited significantly higher desmoplastic and pro‐tumorigenic phenotypes compared with counterparts isolated from women with low mammographic density [117, 299, 300]. Also in breast cancer, tissue‐resident fibroblasts exposed to local secretion of granulin increase the expression of a variety of ECM components [301], promoting the desmoplastic response and malignant progression of otherwise indolent tumours [302, 303]. The increases in ECM reorganisation are catalysed by CAF‐derived MMPs and LOX proteins, which cross‐link collagens and mediate fibre elongation and fibre realignment to promote a stiffer medium conducive with malignant progression [304, 305, 306, 307]. Importantly, CAF‐driven changes to the ECM in the developing TME do not occur in isolation. Instead, reciprocal, dynamic communication exists. Upon remodelling the environment to create a dense, more rigid matrix, fibroblasts sense the modified mechanical cues, in turn stimulating production of cross‐linkers LOX to further stiffen the ECM, signal proliferation, or promote acquisition of a CAF phenotype via the SRC‐YAP‐MYL9/MYL2 axis [218, 308].

Thus, within the developing TME, CAFs evolve to alter cellular, structural and chemical aspects of tissues, but the physical changes they afford through deposition and remodelling of the ECM themselves also play a pivotal role in malignant transition.

### 3.3 Mechanical cues in the developing tumour microenvironment

A gradual shift in the rigidity of the ECM from that of normal tissue is implicated in malignant progression due to its far‐reaching impact on cell morphology, migration, alignment, angiogenesis and immune responses [116, 123, 309, 310, 311, 312]. In addition to diverse paracrine stimuli, epithelial cells respond to physical stimuli to promote proliferation and morphological adaptations conducive with acquisition of cancer traits. The impact of increasing ECM stiffness alone on epithelial behaviour was elegantly shown by Paszek *et al*. in 2005 [309], a concept that was built upon more recently using methacrylated hyaluronic acid hydrogels [312]. Here, Ondeck *et al* dynamically tuned gels from normal (< 150 Pascals) to malignant (> 3000 Pascals) stiffness to demonstrate coincident mechanical sensing and loss of mammary epithelial morphology through TGF‐β and YAP signalling. Changes in stiffness together with increasingly aligned fibres promote tumour cell migration, leveraging traction against remodelled matrix [294, 310, 313]. Indeed, the mechanical coupling of ECM with epithelial cells and CAF cytoskeleton is critical to cell motility in transforming tissues, with benign and malignant cells exhibiting very different mechanical properties [309, 314].

Extracellular matrix tethers and immobilises many soluble mediators, serving as a valuable reservoir for pro‐tumour, CAF‐activating and immune‐suppressive factors such as EGF, FGF, PDGF and critically TGF‐β [119, 296, 315, 316]. Many of these provide subtle guidance cues when tethered, but are fully bioactive once released from proteoglycans upon proteolytic degradation. Moreover, matrix remodelling and resulting increases in stiffness occurring as desmoplasia progresses in developing tumours can support the angiogenic switch. This may be a direct consequence of mechanical compression of vessels driving a hypoxic response [317, 318, 319], through mechanosensing properties of endothelial cells [320, 321, 322], or through MMP‐mediated remodelling via release of matrix‐bound factors such as VEGF, periostin and tenascin C [323]. Disruption of matrix structure, for example inhibition of modifying enzymes such as LOXL family members and reduction in cross‐linked collagenous ECM matrix, has been reported to impair both tumorigenesis and angiogenesis [324].

Remodelling of the ECM modulates immune cell trafficking and functional status [325, 326, 327]. This may occur via direct signals from components of the TME, but also as a consequence of age. Most cancers arise in individuals over the age of 60 [328], and in aged tissues, both fibroblasts and immune populations undergo significant alterations [329, 330, 331]. Altered secretomes, and metabolic and ECM profiles have the potential to combine, propagating pro‐tumour dysfunctional states. An increasingly stiff matrix can induce CAFs to produce chemoattractants such as CCL2 and CSF‐1, attracting innate immune cells, which may deposit further collagen [119]. This ECM also serves a source of DAMPs. Proteolytic cleavage of matrix components such as fibrinogen, fibronectin domains, versican and decorin creates fragments recognised by TLR2 and TLR4 on immune cells [332, 333, 334, 335, 336, 337]. Interactions can promote both differentiation and dysfunction; however, whether such interactions mediate anti‐ vs. pro‐tumour behaviour remains less clear. Across melanoma, lung, colon and liver cancers, versican has been reported to drive an immunosuppressive DC phenotype via TLR2, but has also been reported to direct DCs into an inflammatory phenotype, critical for T‐cell infiltration and antitumour immunity [338].

The physical properties of remodelled ECM exert diverse effects on epithelial, stromal and immune cells it surrounds [116, 310, 339, 340, 341, 342, 343]. The ECM can determine macrophage shape and polarisation [344], and collagen‐rich matrices bias towards pro‐tumorigenic phenotypes, whereas ECM rich in fibronectin promotes antitumorigenic phenotypes [345, 346, 347, 348]. Indeed, increased deposition of type I collagen has been shown to directly stimulate inhibitory receptors such as LAIR‐1 on immune cells [349]. Moreover, YAP activation in cells sensing tensional cues induces the expression of cytokines to recruit MDSCs and TAMs [350]. A modified ECM, rich in fibrillar collagen, further contributes to immune suppression by acting as a physical barrier. T cells are frequently more abundant within ECM‐rich areas surrounding epithelial cells, and such physical exclusion is associated with poor prognosis [216, 325, 351, 352]. Stiffer substrates have been associated with impaired effector function [353, 354]; within dense matrices, T cells are less motile since migration, which is protease‐independent, relies on a rapid ability to deform and squeeze through pores and along fibres [355, 356]. And while impeded, they may experience prolonged exposure to immune‐suppressive cytokines.

Beyond any direct effects on cell behaviour within the developing TME, an accumulation of collagens, increased cross‐linking and stiffening, and rapidly expanding blood vessels lead to a gradual increase in interstitial fluid pressure [317, 319]. This feature itself can contribute to transformation of the local environment. Increases to the movement of fluid through a tissue, called interstitial flow, are sufficient to drive changes in fibroblast and collagen fibres towards a more aligned orientation associated with increasing stiffness [357, 358]. Similarly, fluid movement stimulates the production of TGF‐β by fibroblasts [359] contributing to the alterations in CAF activation status and ongoing immunosuppressive activity. Interstitial flow supports the formation of subtle transcellular chemokine gradients guiding cells out of physical contact with the developing tumour and towards draining lymphatic vessels, which also sense fluid flux, upregulating immune‐homing chemokines and cell adhesion molecules in the process [360, 361, 362, 363]. Such cues are key for efficient immune trafficking, particularly antigen‐presenting cells, to and from the local environment during tumorigenesis.

A multiplicity of mechanisms exists for CAFs to influence, and be influenced by, the developing TME. With growing evidence and technical precision, this is being ascribed to an increasing number of unique CAF subpopulations, defined by tissue‐specific markers. This overwhelming complexity can be distilled, however, into the recurrent functional observations of these CAFs performing either immune‐modulatory, ECM deposition or mechanical remodelling roles. Whether these specialised subpopulations are present from the outset of malignancy as tissue‐resident fibroblasts or whether they are plastic and interconvert with developing cues in the TME remains an open question in the field.

## 4 Outreach activities: systemic effects of the tumour microenvironment

This review has focussed on the reciprocal microenvironment changes that facilitate early tumour growth. However, as practising oncologists are aware, cancer manifests as a systemic illness. The molecular details of how cancer ‘reaches out’ to influence whole‐body physiology are beginning to be understood [364, 365]. Again, much of the focus has been on immunity. Systemic immunity is impaired in patients with advanced cancer, and this is clinically relevant with both success of immunotherapy and mortality from intercurrent infections [366, 367]. Several recent mouse‐based studies have described widespread immune alterations present in cancer [17, 368]. The microenvironment of the tumour draining lymph node changes in parallel to tumour development [269, 369], which may impair onset of immunity or facilitate metastasis. Immune cytokines may also influence bone marrow function to release immature myeloid cells which traffic to the TME to become MDSCs. There has also been interest in these effects as a prognostic or predictive marker, in particular the neutrophil:lymphocyte ratio which is associated with patient outcomes and may be a crude measure of systemic immune dysfunction [370]. In addition to the immunological effects, cancer induces systemic metabolic changes that manifest as cachexia. Intriguingly, this may be mediated by reciprocal systemic stromal changes: Roberts et al. demonstrated that the depletion of CAF‐like fibroblast populations from muscle and fat is responsible for loss of mass in these organs in cancer [371]. There is also overlap between immune and metabolic reprogramming: the inflammatory mediators that induce cachexia in turn elevate immune‐suppressive factors such as cortisol, which further impairs antitumour immunity [372]. It will be fascinating to see how far these systemic changes can be traced to early tumour development.

## 5 Capturing a changing tumour microenvironment, from mice to humans

Historical mouse models of cancer often use injectable human cell lines in immunodeficient mice. While these models have been key to the progress in developing cancer treatments for the past half‐century, they do not model the early microenvironment or the adaptive immune component of the TME. Immune‐based treatments are now central to the practice of oncology, and so, it is essential to use immune‐competent models that reflect the whole TME. Advances over the last decade in genetic engineering have increased the feasibility of spontaneous tumour models, which have a neoantigen profile and local immune infiltrate more representative of human cases than injectable tumour models [373, 374, 375, 376]. However, 65 million years of evolution have resulted in a number of significant mechanistic differences between mice and humans, for example Ly49 vs. killer immunoglobulin receptor expression on NK cells [377], differing antibody isotypes [378, 379] and differing transcriptional programmes for B‐cell and T‐cell development [378]. There are also genomic differences between mice and humans that complicate developing spontaneous models.

Concerted efforts are now being made to ‘humanise’ murine models as far as reasonably possible. Patient‐derived xenografts (PDX) in combination with an immune system reconstituted from an autologous haematopoietic stem cell are increasingly used in tumour immunology, especially to predict patient response to therapy [380]. However, the injection of large numbers of late‐stage cells to create a viable xenograft limits their use for the study of the early TME. Organoid technology meanwhile maintains many of the advantages of PDX, including maintaining heterogeneity [381] and tumour–stroma interactions [382, 383], while also allowing for high‐throughput investigation of the earliest stages of tumour development. For example, it was shown that extended TNF‐α treatment of human ovarian surface epithelial cell‐derived organoids led to loss of normal structural organisation and development of an early cancer legion, thus suggesting a link between a pro‐inflammatory microenvironment and the initiation of tumorigenesis [384]. Similarly, a library of human CRC organoids was created from a range of tumour stages including normal epithelium up to metastatic cancer. Sequencing of each organoid highlighted a high dependency on culture ‘niche factors’ was present in early organoids, and a subsequent mutational loss of this requirement was a critical stage in development into an adenocarcinoma. The authors then went on to validate this suggestion that transition from early cancer to invasive cancer requires increased growth factor autonomy, xenografting the organoids onto mice [385]. It is becoming increasingly clear therefore that improved understanding of the early TME will require utilisation and expansion of both the *in vivo* and *in vitro* toolkit (Table 1).

**Table 1:** Advantages and limitations of mouse models in the early TME.

Model system	Advantages	Limitations
Subcutaneous xenograft (cell line or patient‐derived)	Rapid turnaround of experiments, high throughput Human cancer cells Tractable: genetic manipulation of cell lines to study pathways accessible	Does not capture organ‐specific or early TME Requires immunocompromised mice Replacement of stroma by host
Subcutaneous mouse cell lines	Rapid turnaround of experiments, high throughput Tractable: genetic manipulation of cell lines to study pathways accessible Intact immune system	Does not capture organ‐specific or early TME Species differences in immunity Highly transformed cells not representative of early stages or heterogeneity
Orthotopic injection models	Models the organ‐specific tumour microenvironment If mouse cells used, intact immunity	Injection of highly transformed cells, large numbers, does not model early TME
Genetically engineered mouse models	Models full development of lesions, including premalignant stages Intact immune system Accurate modelling of organ‐specific and early microenvironment	Expensive and time‐consuming Specialised techniques needed for tumour monitoring ‘Clean’ – defined oncogenic drivers decrease mutational spectrum Mouse genomic differences mean mutating homologs of human oncogenes does not necessarily produce the same organ cancer Mouse immune differences may be significant Timescales of months still do not match the many years of tumorigenesis in humans Not available for all cancers yet
Humanised Mouse models	Allows study of human cancer‐immune interactions in an animal system May overcome species differences in immunity	Expensive and time‐consuming Transplantation of transformed tissue, which may not reflect early lesions Stromal elements are mouse‐derived Marrow transplantation may produce off‐target graft vs. host effects

## 6 Early detection and therapeutic intervention

For many cancers, early detection and intervention significantly improve survival. In oesophageal cancer, the detection of the premalignant condition Barrett's oesophagus can dramatically increase dismal 5‐year survival rates of less than 20%. However, in the case of Barrett's, monitoring by endoscopic surveillance represents a significant cost and disruption to patients' lives. Thus, identifying the minority that will go on to develop OAC is a priority. Examining the underlying microenvironment may help to define and stratify patients at greater risk. Indeed, molecular imaging has been used to exploit changes in tissue glycosylation for the identification of dysplastic tissues [386, 387, 388]. Early detection of cancers also requires the discovery and use of robust biomarkers to identify lesions likely to progress. Thus, strategies to screen at‐risk groups in a minimally invasive fashion are increasingly focusing on analysis of the premalignant lesion. Although currently focused on detecting early changes in the epithelial compartment [389, 390], our increasing understanding of the complex interactions at play within tissues undergoing malignant transformation presents the opportunity to exploit changes within the surrounding stroma for early detection and patient stratification (Table 2). Changes in immune content and localisation [18], ECM characteristics [391, 392, 393] and functional gene signatures [145, 394, 395] all offer potential. Indeed, sequencing approaches have enabled large‐scale, single‐cell resolution characterisation of the immune landscape of over 30 different tumour types [396].

**Table 2:** Pro‐ and antitumour hallmarks of the early TME. SHH, sonic hedgehog; TKI, tyrosine kinase inhibitors.

Function	Stromal mechanism	Therapeutic strategies
Antitumour early functions		
Immune surveillance of nascently transformed epithelial cells	**T cells** – *cytokines:* TNFs, IFN‐γ, granzyme B, *receptors:* FasL, *cell contact:* perforin, granzyme B. **NKs** – *cytokines:* TNFs, IFN‐γ, CSF‐2, granzyme B, *receptors:* NKG2D, KIRs, KKp44, *cell contact:* perforin. **ILCs** – *cytokines:* TNFs, IL‐2, IL‐8. *Neutrophils* – *cytokines:* IFNs TNF‐⍺, NO, H_2_O_2_, *receptors:* TRAIL, costimulatory molecules	ICPI (PD‐1/PD‐L1, CTLA‐4, TIM3, LAG3) Vaccines that prevent initial transformation (HPV) CAR T and NK cells Microbiome modification
Recruitment of tumour neoantigen‐specific T cells	**CAFs** – *cytokines:* TNF‐⍺, IL‐1β, *chemokines:* CXCL9, CXCL10, CXCL11, *DAMPs, matrix remodelling*. **T cells** – *cytokines:* IFN‐g, TNFs. **Macrophages** – *chemokines:* CXCL9, CXCL10, CXCL11, *DAMPs, matrix remodelling*. *DCs* – *chemokines*: CCL2, CCL3, CCL17, CCL21, XCL1, *receptors:* costimulatory receptors, MHC	ICPI Chemokine modulators (e.g. CXCR4/CXCL12 axis) Engineered DC‐based vaccines (Sipuleucel‐T)
Promotion of ‘M1’ macrophages	**CAFs** – *cytokines:* VEGF, *receptors:* ICAM‐1, **chemokines** – CCL2, CCL9, DAMPs. **T cells** – *cytokines:* TNF‐⍺, IFN‐γ, *chemokines:* NO, ROS, IL‐2, DAMPs. **Macrophages** – *cytokines:* IFN‐γ, DAMPs. **Neutrophils** – cytokines: MIPs, IFN‐γ, DAMPs	Myeloid‐modulating therapies (TKI/ anti‐VEGF, CD47/SIRPA axis) Depletion/repolarisation of M2 macrophages Radiotherapy (release DAMPs to promote innate response)
Pro‐tumour early functions: impact on cancer cells		
Sustained proliferative signalling and transformation	**CAFs** – *cytokines*: TGF‐β, IL‐1b, IL‐6, *growth factors*: HGF, IGF, EGF, LIF, oncostatin M, FGF, exosomes, MMPs. **ECM** – *growth factors*: following matrix degradation, increases in stiffness. *mutagens*: ROS, NOS. **TAMs** – *growth factors*: TGF‐β, EGFs, VEGF, PDGF, MMPs. **TANs** – *cytokines*: TGF‐β, MMPs **ILCs** – *cytokines*: IL‐22	Growth factor inhibitors (e.g. anti‐EGFR: cetuximab) TKI (axitinib,regorafenib, lenvatinib) ECM modulation – PEGPH20, losartan, simtuzumab, MMP inhibitors, cytokine/ chemokine blocking
Resisting cell death	**CAFs** – *cytokines:* TGF‐β, IL‐6, IL‐10, IL‐11, *receptors*: PD‐L1, FasL, matrix deposition. **T cells** – *receptors*: reduced TRAIL, FasL. **TAMs** – *cytokines*: IL‐6, IL‐11, TNF‐⍺, matrix deposition. **TANs** – *cytokines*: IL‐6, IL‐11. **ILCs** – *receptors*: reduced TRAIL, FasL	ICPI Pro‐apoptotic activators Anti‐apoptotic inhibitors
Pro‐tumour early functions: impact on stromal cells		
Metabolic support of growing tumour	**CAFs** – *cytokines*: TGF‐β *metabolites*: alanine, glutamine, lactate. **Endothelial cells** – angiogenic switch. **TAMs** – *metabolites:* IDO, Arg1, lactate, angiogenic switch	Metabolite inhibitors Vascular ‘normalisation’ (avastin) Fibroblast ‘normalisation’ Reprogramming of TAM
Recruitment of immune‐suppressive cells	**CAFs** – *cytokines*: C3, CSF, IL‐6, IL‐10, TGF‐β, GDF‐15, *chemokines*: CXCL12 and CXCL16. **Endothelial cells** – *receptors*: CLEVER‐1. **T cells** – conversion to T_regs_. **TAMs** – conversion to MDSCs, *cytokines:* IL‐6, IL‐10 and TGF‐β, *chemokines*: CCL5, CCL17, CCL22, *metabolites*: IDO, arginase. **ILCs** – conversion to ILC2 and ILC3	ICPI Neutralising antibodies Application of decoy receptors or chemotraps
Impairing T‐cell activity	**CAFs** – *cytokines*: TGF‐β and IL‐6, *metabolites*: PGE2 and IDO, *receptors*: PD‐L1, PD‐L2 and FasL, inducing fibrosis. **T cells** – *cytokines*: TGF‐β, reduced pro‐inflammatory cytokines, *receptors*: PD‐L1, CTLA‐4, Fas, LAG3, TIGIT, conversion to T_regs_. **Macrophages** – *cytokines:* IL‐10, *chemokines*: CCL22, *metabolites*: IDO, Arg1, lactate, *receptors*: PD‐L1, inducing fibrosis. **DCs** – *cytokines*: TGF‐β, IL‐10, *metabolites*: IDO, Arg1 *receptors*: PD‐L1, PD‐L2, reduced costimulation. **ILCs** – *cytokines*: IL‐12, IL‐22, *receptors*: NKp46	ICPI Tumour vaccines Adoptive transfer: TIL therapy, TCR engineered cells and CAR T cells Immune‐stimulatory treatments (STING pathway)
Fibroblast function	**Fibroblasts** – *cytokines*: TGF‐β, IL‐1a, *growth factors*: PDGF‐2,FGF‐2, microRNA **ECM** – increases in stiffness, *signalling: YAP‐TAZ, HSF‐1* **T cells** – *cytokines:* IL‐1, IL‐6 and TNF. **TAMs** – *cytokines:* TGF‐β, IL‐6, growth factors: PDGFs, FGF2, *mutagen*: ROS	Prevent CAF activation (SHH inhibitor saridegib, galunisertib) CAF action (AMD3100) CAF ‘normalisation’ (ATRA, paricalcitol) Destabilisation of ECM Reprogramming of TAM

Developing our comprehension of how immune and fibroblastic components contribute to the transition from premalignant to cancer affords a range of clinical approaches for early intervention. Understanding kinetics of the host responses in malignant transition, such as loss of immune surveillance, may provide a unique therapeutic window. Preclinical models have explored strategies to prevent or reverse the switch in innate cells to maintain or reinstate an antitumour phenotype. However, while attractive, many pathways disrupted during immune suppression are key to maintain immune tolerance and protection against autoimmunity, thus global approaches to inhibit the innate immune system in early cancer may be counterproductive. Instead, due to their diverse array of functions, targeting the fibroblasts may provide an alternative method to stall malignant transition. While eradication strategies have been investigated, such an approach may in practice be hampered by the lack of specific markers and complexities stemming from heterogeneous pro‐ vs. antitumour subsets [214, 216, 232, 233]. Reprogramming or ‘normalisation’ methods are proving more attractive. Off the back of studies identifying novel stromal targets, methods to interfere with CAF activation status and improve traits such as mechanical properties via inhibition of factors including FGFR, hedgehog, NOX, TGF‐β, LOX, hyaluronic acid and all‐trans retinoic acid (ATRA) pathways have progressed from preclinical models to clinical trial. Utilisation of re‐normalising the TME for enhancement of existing therapy is well‐demonstrated by a recent report in murine breast cancer. By modifying an angiotensin blocker to be pH‐dependent, the drug was active in the more acidic microenvironment of a developing tumour but not in normal neutral pH tissue. Therefore, only CAFs in the TME were reprogrammed to a normalised state, and not activated fibroblasts in adjacent tissue. This normalisation enhanced the efficacy of ICPI. Likewise, the repurposing of existing agents shown to have effects on stroma and wider TME is under trial [397, 398, 399, 400]. For example, in PDAC, the drug ATRA (which is widely used to treat acute promyelocytic leukaemia) was shown to restore pancreatic stellate cells to a quiescent state only seen in the very early stages of tumour initiation, and these treated cells displayed significantly decreased pro‐tumour functions. However, the ability of mouse models to predict therapeutic effect is not straightforward. Preclinical models suggest that hedgehog inhibitors may be useful for the treatment of pancreatic cancer by restricting stromal growth and improving drug penetration [401]. However, subsequent clinical trials have been disappointing [402]. Further preclinical studies have shown that while hedgehog inhibition reduces desmoplasia, it can also enhance epithelial growth. The unpredictable balance of these factors in humans may then determine whether the treatment has a positive or negative effect [403].

Beyond the design of novel therapeutic targets, a thorough understanding of the composition and roles of stroma of the early tumour microenvironment may inform patient prognosis or predict response to treatment [404, 405, 406]. For example, a stromal gene signature has been shown to predict poor survival and metastasis in high‐grade serous ovarian cancer. In summary, although the early stroma offers many opportunities for intervention for patient benefit, key barriers to advancing this are the early detection of these lesions, and a more thorough understanding of the relationship between animal models and different human tumours at early stages of development.

## 7 Perspectives and conclusions

Tissues surrounding mutated cells play a pivotal role in carcinogenesis, from initial elimination responses through to supporting growth and development of a permissive niche. Recent technological advances have dramatically increased our appreciation of the complex networks at play, the duality of our immune system, the increasing diversity and functional specialisation of fibroblasts, and the impact of changing tissue mechanics. Even so, outstanding challenges remain. We know relatively little about the plasticity, heterogeneity and interdependencies that define the evolving stromal landscape. Nevertheless, it is likely that these complex tumour–stroma interactions compliment the well‐documented tumour heterogeneity and cumulatively contribute to increased robustness of early tumours [407], and we now face the challenge of dissecting these roles, addressing impact of tissue specificities and previously overlooked traits such as the ageing microenvironment. A better understanding facilitated by the development mouse models that more faithfully recapitulate early stages of cancer, the installation of extensive patient sample repositories and inclusion of stromal parameters in trials will help to unravel underpinning mechanisms in real time, and most importantly stand to inform new approaches to better identify and treat patients early.

## Conflict of interest

The authors declare no conflict of interest.

## Author contributions

JDS conceived the review, and WMM, JOJ and JDS wrote the paper.
